# Bibliometric analysis of treatment modalities in calcific aortic valve stenosis

**DOI:** 10.3389/fphar.2025.1431311

**Published:** 2025-03-13

**Authors:** Yang He, Yue-Jiao Yang, Zhao-Jun Wang, Liang Tang

**Affiliations:** Department of Cardiovascular Medicine, The Second Xiangya Hospital, Central South University, Changsha, Hunan, China

**Keywords:** calcific aortic valve stenosis (CAVS), research hotspots, knowledge mapping analysis, treatment modalities, bibliometric analysis

## Abstract

**Background:**

Calcific aortic valve stenosis (CAVS) is a common cardiovascular condition associated with significant adverse events and high mortality rates. Unfortunately, there are currently no effective pharmacological treatments to halt or prevent its progression. Through our analysis of global trends and treatment strategies, we have identified valuable insights and promising therapeutic possibilities. Additionally, by utilizing bibliometric and visualization techniques, we provide a comprehensive overview of the current research landscape in this field.

**Method:**

According to our design idea, we used the Web of Science database to select publications on aortic stenosis and related treatments. Through our VOSviewer and CiteSpace analysis, a total of 787 articles have been analyzed by September 2024. We also summarize and explore the most prolific authors, the most prolific countries, and the journals and institutions that publish the most articles.

**Results:**

A visual analysis of the collected articles reveals that Canada and the United States have the highest publication volumes in this field. Among institutions, Harvard University in the U.S. leads in publication count, followed by Laval University in Canada and the University of California in the U.S. The top three research hotspots are stenosis, calcification, and progression. The journal with the highest number of publications in this area is Frontiers in Cardiovascular Medicine, followed by Catheterization and Cardiovascular Interventions and Arteriosclerosis, Thrombosis and Vascular Biology. Furthermore, research on CAVS treatment spans various directions and focuses, including therapeutic approaches, pathogenesis, and diagnostic methods.

**Conclusion:**

Research into CAVS treatment has advanced significantly over the years. While interventional and surgical valve replacement remains the mainstay treatments for aortic stenosis, they are insufficient to fully meet the needs of the patient. Emerging priorities now focus on improving diagnostics, exploring innovative therapies, uncovering disease mechanisms, and developing novel drugs. These findings highlight the evolving demands in this field and underscore the need for continued research to address these challenges.

## 1 Introduction

Calcific aortic valve stenosis (CAVS) is one of the most prevalent valvular heart diseases, primarily caused by the calcification of the aortic valve. This pathological process is closely associated with aortic stenosis and the onset of various cardiovascular complications (Kraler et al., 2022; Lindman et al., 2016). Over the past two decades, our understanding of CAVS has significantly evolved, challenging the long-standing belief that it is merely a degenerative condition (Otto, 2008; Rajamannan et al., 2007; Yip and Simmons, 2011). Emerging research has revealed a paradigm shift, suggesting that CAVS is an active pathological process sharing common risk factors and pathophysiological features with atherosclerosis (Zebhi et al., 2021; de Oliveira et al., 2020; Stewart et al., 1997; Kostyunin et al., 2020). The progression of aortic stenosis is now recognized as occurring in two distinct phases: an initial stage characterized by valvular lipid deposition and inflammation, followed by a later phase dominated by pre-calcific and osteogenic processes (Diederichsen et al., 2022). Calcification of the aortic valve is a highly complex and protracted process, driven by an imbalance between promotive and inhibitory factors regulating calcium deposition on the valve (Iqbal et al., 2023).

In recent years, therapeutic research into valvular stenosis has intensified, leading to the exploration of various drug trials and innovative therapies. Currently, treatment remains primarily centered on surgical aortic valve replacement (SAVR) and transcatheter aortic valve replacement (TAVR), both of which are invasive and impose significant burdens on patients (Moncla et al., 2023). To address these limitations, current research is dedicated to advancing drug development, technological innovation, and mechanistic understanding. Drug therapies, which are generally well-accepted by patients, have been the focus of extensive trials involving agents such as simvastatin (Rossebø et al., 2008), vitamin K (Diederichsen et al., 2022), denosumab (Pawade et al., 2021). However, no clear therapeutic benefits have been demonstrated to date. Novel approaches, such as small RNA therapies and ultrasound-based treatments, present promising options, especially for patients with severe stenosis (Duarte and Giugliano, 2022). Over the past two decades, research has increasingly focused on the underlying mechanisms of valvular stenosis, including inflammation, lipid metabolism abnormalities, and oxidative stress—each identified as a potential therapeutic target.

Bibliometrics, an interdisciplinary field, applies mathematical and statistical methods to quantitatively analyze knowledge dissemination. Originating in the early 20th century and formalized as a discipline by 1969, bibliometrics integrates mathematics, statistics, and documentation to construct a comprehensive quantitative framework (Thelwall, 2008). By employing these methods, bibliometric analysis facilitates detailed evaluations of existing literature, offering comprehensive insights into authors, keywords, journals, countries, institutions, and references (Kokol et al., 2021; Merigó and Yang, 2017). This type of analysis uncovers the developmental trajectory, current state, key focus areas, and emerging trends within a field. Furthermore, information visualization tools complement this process by providing structured and detailed representations of the field’s dynamics (Begum et al., 2018; Ding and Yang, 2022).

Despite extensive research into aortic stenosis treatment, thus far, no bibliometric analysis has systematically summarized the development, research hotspots, and scientific trends specifically in the field of CAVS. Therefore, in the present study, we aim to conduct a comprehensive bibliometric analysis to fill this gap and provide a detailed overview of the research landscape, key areas of focus, and the emerging trends in CAVS.

## 2 Methods

### 2.1 Research tools

Citespace and VOSviewer are pivotal tools in the domain of bibliometric analysis and mapping. Despite their distinct emphases and individual merits, they synergize harmoniously, offering complementary functionalities. Citespace primarily focuses on document visualization and analysis, proficiently converting literary data into visual representations such as author co-occurrence networks, citation relationship networks, and topic evolution maps. These visual aids facilitate researchers’ comprehension of the intricate relationships and evolving trends within the literature (Abramo et al., 2011). The fundamental concept behind VOSviewer is co-occurrence clustering, which identifies relationships between two entities based on their simultaneous appearance (Ding and Yang, 2022). These correlations can exhibit various types, strengths, and directions. Through the utilization of measurement indices to cluster these relationships according to their strength and direction, VOSviewer facilitates the identification of distinct groups within the literature landscape (Ding and Yang, 2022).

### 2.2 Data collection

The Web of Science (WoS) serves as a premier digital literature resource database and retrieval platform, facilitating efficient identification and access to pertinent articles. It is widely utilized by a large community of scientists and is recognized as the most suitable tool for bibliometric analysis (Ding and Yang, 2022), literature searching together with other high-quality databases such as PubMed, and Scopus might minimize selection bias. However, bibliometrics cannot screen and sort the data of the given databases at the same time, searching multiple databases simultaneously might lead to a large number of duplicates, resulting in problems in subsequent analysis, so only WoS database is selected for current analysis.

**FIGURE 1:**
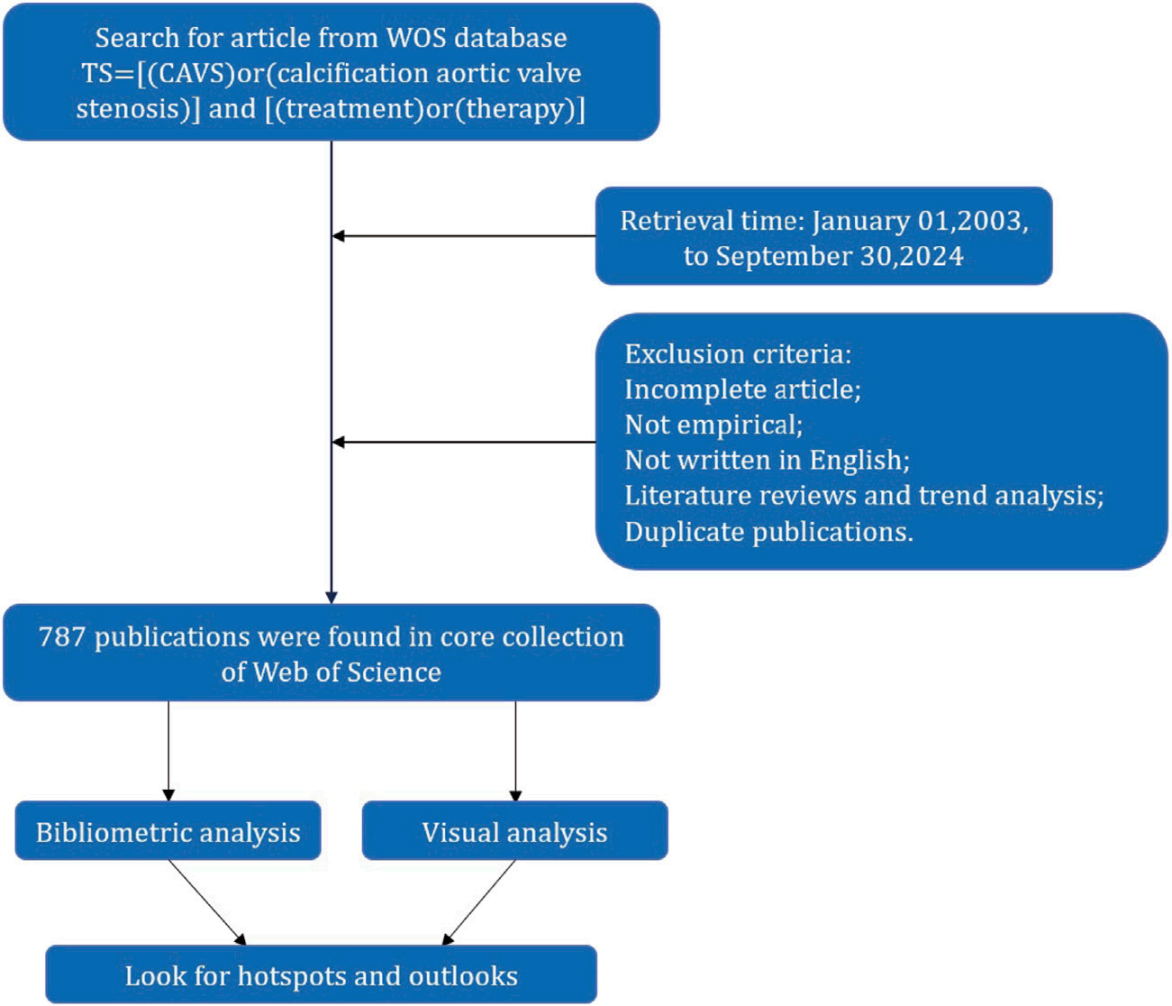
Flowchart of data collection and research procedure.

The data for the present were extracted from the Web of Science Core Collection database, encompassing articles concerning the treatment of CAVS published between January 2003 and September 2024. Subsequently, a bibliometric analysis was performed to quantitatively assess the research outcomes. The search formula was set to TS = [(CAVS) or (calcification aortic valve stenosis)] and TS = [(treatment) or (therapy)] from 1 January 2003, to 30 September 2024. The literature types were restricted to articles and review articles, and after excluding the search results, a total of 787 articles were obtained (Figure 1).

## 3 Result

### 3.1 Chronological map of the literature

**FIGURE 2:**
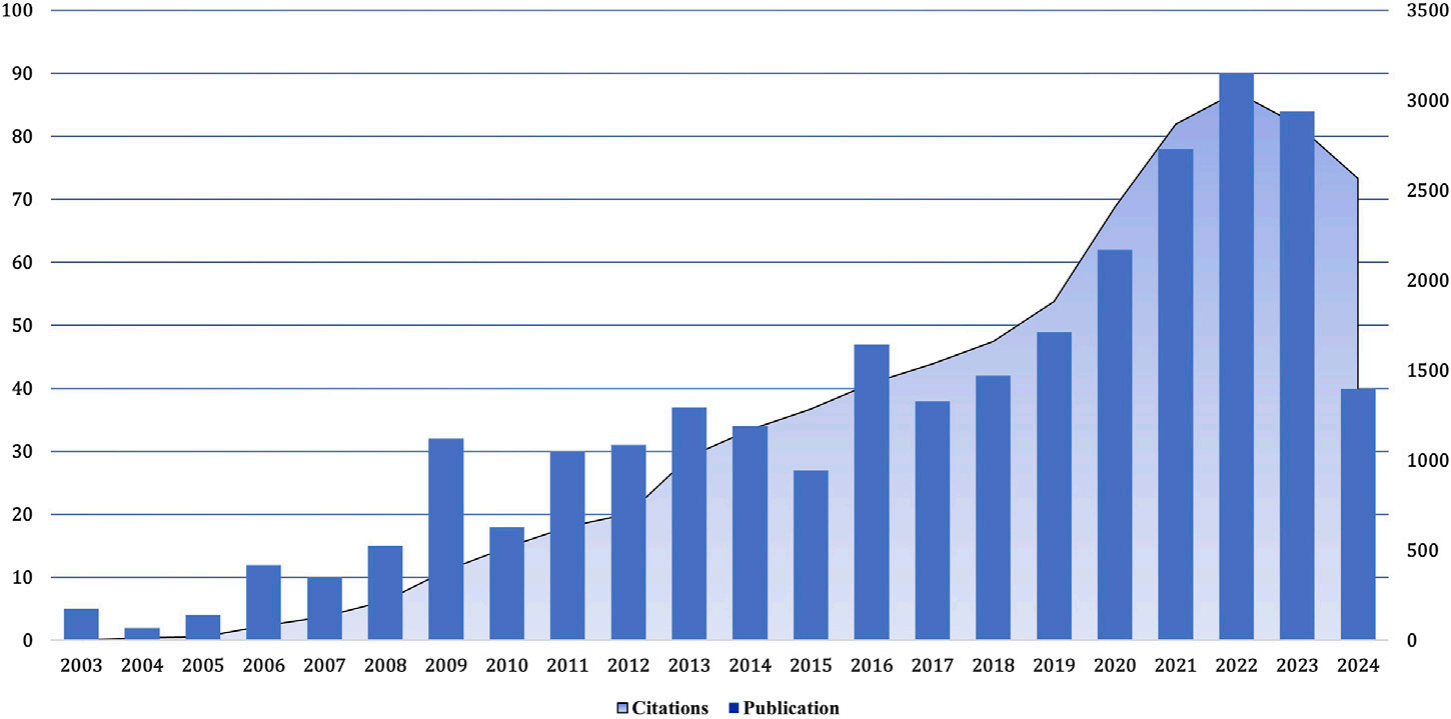
Growth trend of the number of publications and cited papers globally from 2003–2024.09. The number of research papers on the treatment of calcified aortic stenosis rose from 2017 to 2022, and the number of citations increased, especially in 2022.

Between January 2003 and September 2024, there has been a noticeable increase in the number of articles about the treatment of aortic stenosis and related research (Figure 2). The number of published articles shows a steady increase from 2004 to 2006, 2008 to 2009, 2010 to 2013, and 2017 to 2022. However, there were minor fluctuations, with a decrease observed in publications during 2007, 2010, 2014, 2015, and 2017.

Notably, between 2021 and 2023, a notable surge in publications was evident. Nonetheless, due to incomplete search coverage, both publication volume and citation counts experienced a decline in 2023. In 2022, the frequency of citations of relevant articles reached the highest level, and the most cited article was published in 2012. Overall, the number of publications in this field is constantly increasing, and there has been a rapid development stage since 2015, with more than 80 articles published annually in recent years.

### 3.2 National and regional distribution of literature

**FIGURE 3:**
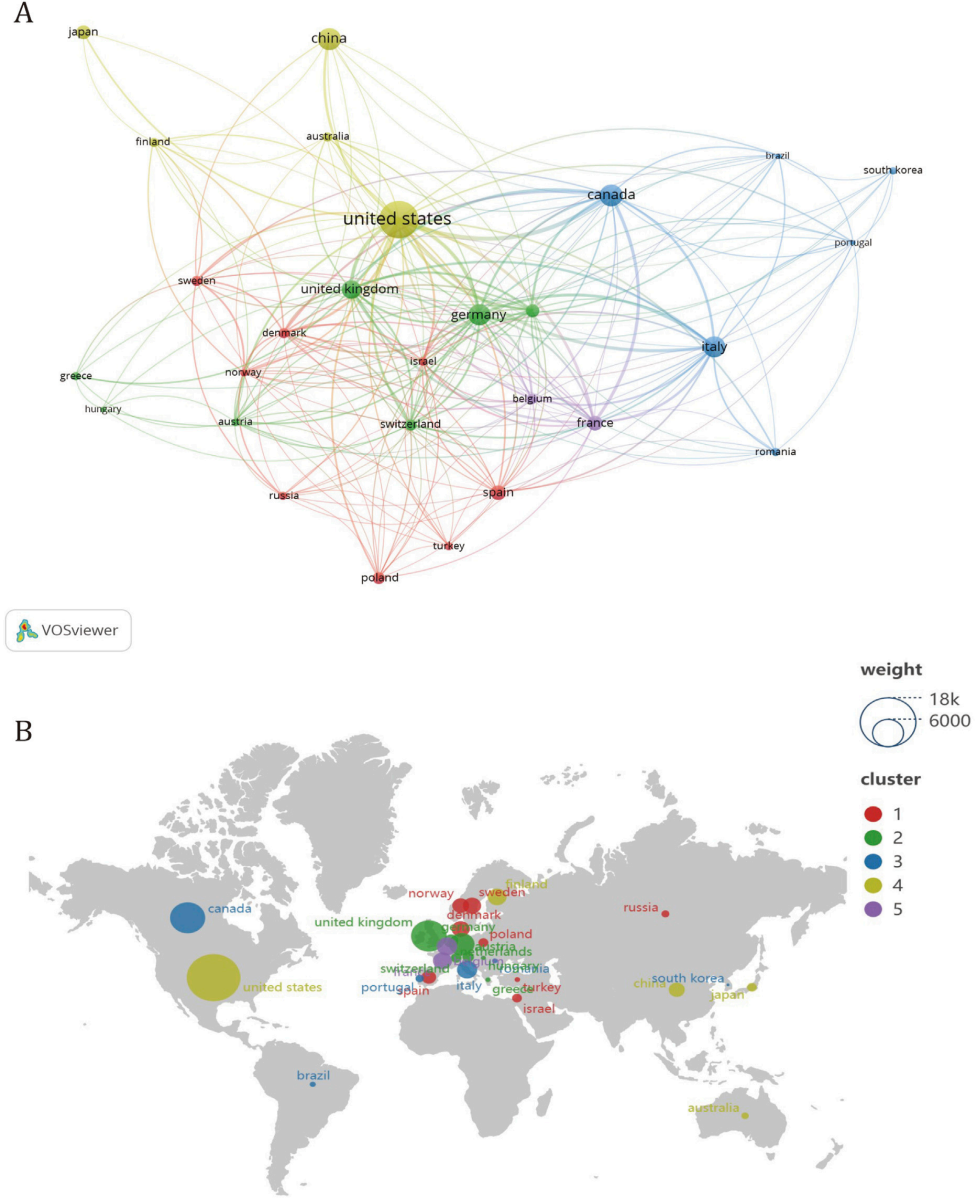
Analysis of cooperation between countries in CAVS treatment. **(A)** Different colors represent countries that cooperate with each other, the size of the circle is proportional to the total number of articles in the corresponding country, and the distance between two countries is inversely proportional to the degree of cooperation between countries. Among them, four different regional groups work closely together. **(B)** The country in the circle is positively related to the average citation rate, and the same article may have co-authors. Among them, the United States is the most cited country.

To ascertain the leading contributors in the realm of treating calcific aortic stenosis, this study scrutinized the publication output across 56 countries. Initially, countries with a publication volume of five or more were visualized using VOSviewer. The resultant visual representation, as depicted in Figure 3A, illustrates various attributes: the size of the circle node correlates with the number of publications it represents; the node connections denote the strength of cooperation, with thicker lines indicating more frequent collaboration between countries; and the node colors signify different cooperative regions. The distribution of publishing countries in this field exhibits considerable disparity, with pronounced prominence among a select few nations. Furthermore, the majority of publications emanate from a handful of countries, accentuating the notable concentration of research efforts within specific geographical regions.

In addition to quantitatively analyzing the output of literature, analyzing established collaboration links not only reveals existing successful collaborations but also indicates opportunities to enhance ongoing collaborations and initiate new partnerships. Based on the analysis of the co-authored country cooperation map (Figure 3A), there are five distinct clusters of national regional cooperation. The United States collaborates closely with countries such as China and Japan, while Canada enjoys stronger collaboration with countries such as Italy, South Korea, and Brazil. The United Kingdom has more cooperation with countries such as Germany, the Netherlands and Australia. Denmark has more collaboration with Norway, Poland, and Russia. France cooperates with countries such as Israel and Spain.

In terms of intercontinental collaboration, the United States exhibits a predominant engagement with numerous countries, notably Canada, Germany, and the United Kingdom. Conversely, among leading Asian nations, such as China, Japan, and South Korea, collaboration appears less robust. Overall, intercontinental partnerships demonstrate greater strength compared to intracontinental ones, with connections among countries within the same continent, particularly within Asia, appearing relatively weaker.

Table 1 lists the top five countries in calcific aortic stenosis research by publication count. Among them, the United States stands out with 267 publications, contributing roughly one-third of all articles in the field. Canada, while second in publication count with 94 articles, shows notable influence, achieving 6,571 citations and an average of 69.90 citations per paper. Interestingly, Figure 3B illustrates that although some European countries have lower publication numbers and are not listed in Table 1, their papers receive relatively high average citations.

**TABLE 1:** Top 5 productive countries regarding the research on treatment in aortic valve stenosis.

Rank	Country	Publications	Citations	Average citation/publication
1	United States	267	15319	57.37
2	Canada	94	6571	69.90
3	China	90	1392	15.47
4	Germany	88	3442	39.11
5	Italy	87	2157	24.79

### 3.3 Map of authors and institutions

Table 2 shows that Philippe Pibarot and Patrick Mathieu from Laval University are the top two authors in terms of the number of publications. Among the top 10 authors in terms of publications, four Canadian authors, all from Laval University, occupy three of the top five positions, while the top three authors in terms of H-index are Philippe Pibarot from Laval University, David Newby from the University of Edinburgh and Sotirios Tsimikas from the University of San Diego, California.

**TABLE 2:** Top 10 authors in the field of CAVS treatment research.

Rank	Author	Country	Institution	Total publications	Citations	H-index	Total link strength
1	Philippe Pibarot	Canada	Laval University	28	2307	113	98
2	Marie-Annick Clavel	Canada	Laval University	19	1586	62	76
3	Patrick Mathieu	Canada	Laval University	18	1284	62	63
4	Elena Aikawa	United States	Harvard Medical School	17	1543	80	17
5	Marc Dweck	United Kingdom	University of Edinburgh	16	1271	66	32
6	Sotirios Tsimikas	United States	University of California San Diego	14	1501	91	37
7	Nianguo Dong	China	Huazhong University of Science and Technology	12	316	33	17
8	David Newby	United Kingdom	University of Edinburgh	12	1052	108	30
9	Romain Capoulade	France	University of Nantes	11	902	33	52
10	Yohan Bossé	Canada	Laval University	10	639	52	47

**FIGURE 4:**
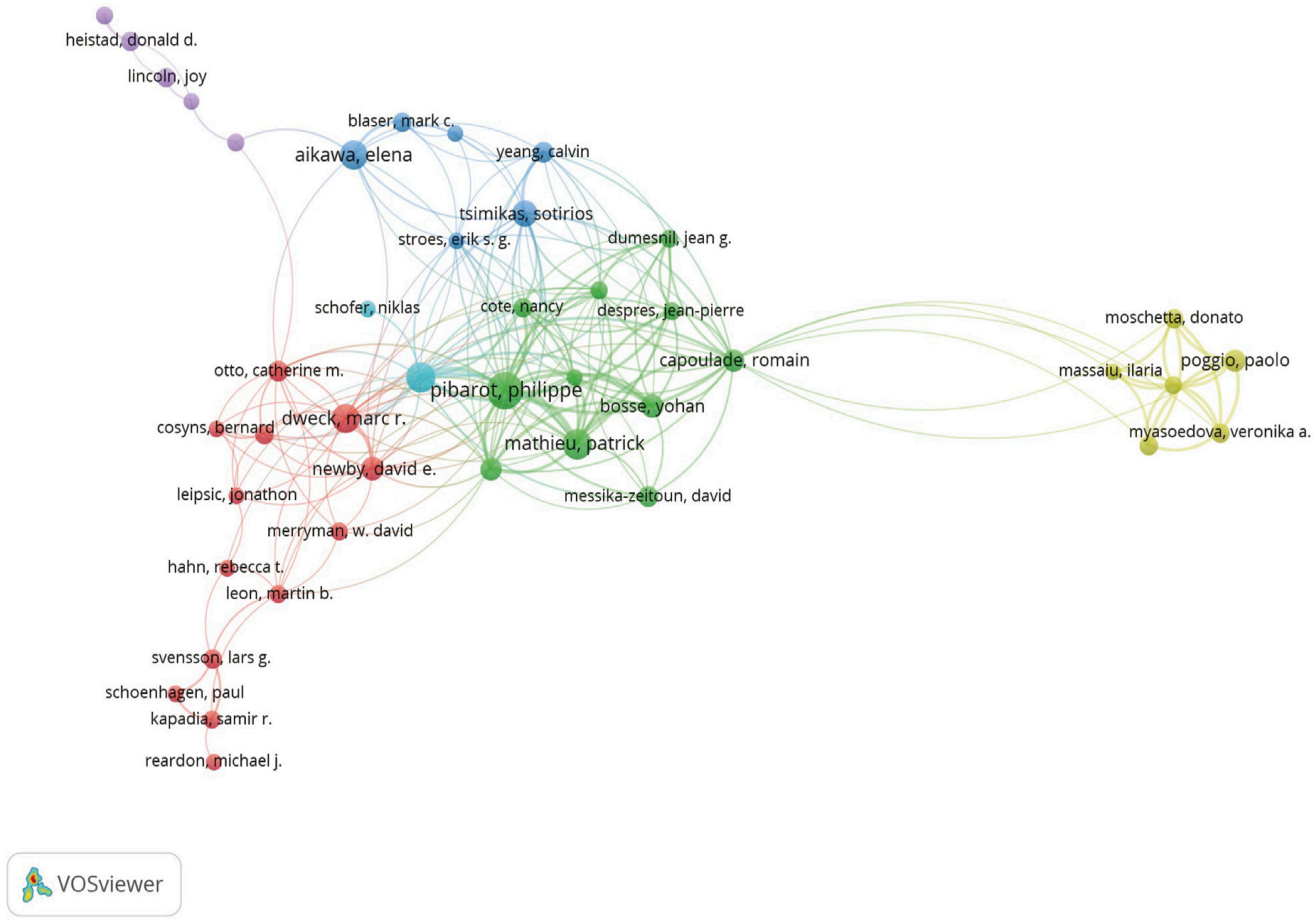
Different colors represent different authors who collaborate with each other, the size of the circles is proportional to the total number of articles by that author, and the distance between two authors is inversely proportional to the degree of collaboration between them.

We clustered authors with five or more publications, analyzing a total of 83 authors. After excluding authors with minimal collaboration, we obtained Figure 4, which shows a strong collaboration between Philippe Pibarot and Patrick Mathieu within the same institution, as well as between Marc R. Dweck and David E. Newby. Additionally, there is a notable collaboration between Elena Aikawa and Sotirios Tsimikas across different institutions.

As shown in Table 3, the institution with the highest number of publications in the field of research on the treatment of calcific aortic stenosis is Harvard University with 47 publications, followed by Laval University with 42 publications, and the University of California with 39 publications in third place. In terms of citations, the Mayo Clinic had the highest ACI (148.35), followed by the University of Washington (126.21) and the University of Edinburgh (106.33).

**TABLE 3:** Top 10 institutions in the field of CAVS treatment research.

	Institution	Country	Quantity	ACI	STC	H-index
1	Harvard University	United States	47	54.7	2571	23
2	Laval University	Canada	42	68.88	2893	26
3	University of California	United States	38	74.47	2830	21
4	Harvard Medical School	United States	36	60.44	2176	20
5	Brigham and Women’s Hospital	United States	32	64.09	2051	19
6	Institut national de la santé et de la recherche médicale	France	22	34.64	762	11
7	University of Edinburgh	United Kingdom	21	106.33	2233	14
8	Mayo Clinic	United States	20	148.35	2967	12
9	Québec Heart and Lung Institute	Canada	19	86.53	1644	16
10	University of Washington	United States	19	126.21	2398	15

ACI, average citations per-item; STC, the sum of the times cited.

**FIGURE 5:**
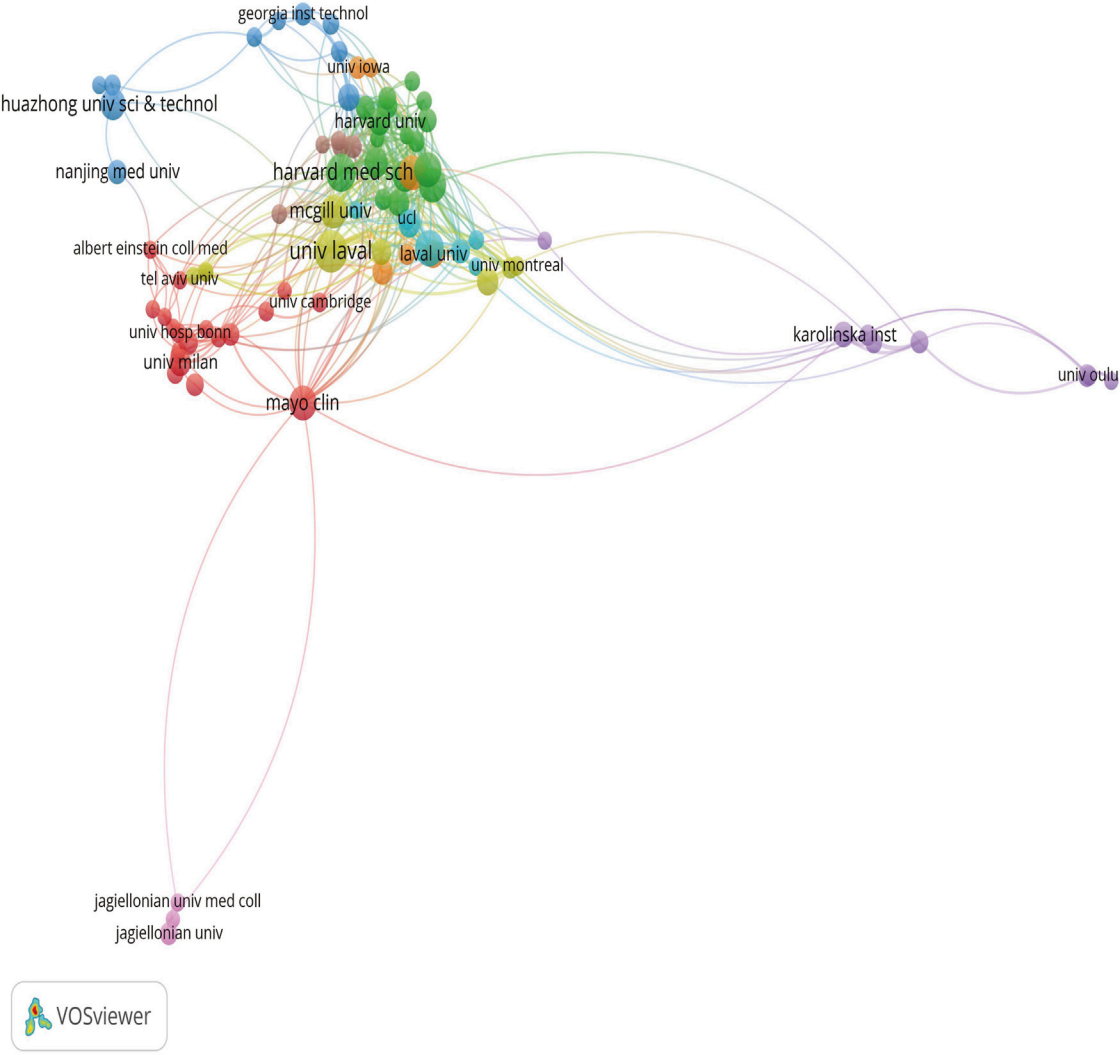
Different colors represent different organizations that want to work with, the size of the circle is proportional to the number of publications issued by that organization, and the distance between two organizations is inversely proportional to the degree of cooperation between them.

Figure 5 shows a set diagram of cooperation between institutions, where different colors can be divided into different sets. The more connections an institution extends, the closer it cooperates with other institutions.

### 3.4 Distribution of relevant literature

The three disciplines with the highest number of publications were Cardiovascular (51.59%), Peripheral Vascular Disease (11.31%), and General Internal Medicine (7.24%), with Cardiovascular accounting for half of all publications. Other disciplines covered in the literature include Pharmacology Pharmacy (6.23%), Surgery (6.10%), Radiology Nuclear Medicine Medical Imaging (4.57%), Hematology (4.19%), and other disciplines spanning the spectrum of medicine and surgery and ranging from molecular basis to clinical application. All of this suggests that this research area is broad in scope, diverse in approach, and a current research hotspot (Table 4).

**TABLE 4:** The top 10 discipline categories in the field of CAVS therapeutic research.

Rank	Quantity	WOS categories	Percentage (%)
1	406	Cardiac Cardiovascular Systems	51.59
2	89	Peripheral Vascular Disease	11.31
3	57	Medicine General Internal	7.24
4	52	Biochemistry Molecular Biology	6.61
5	49	Pharmacology Pharmacy	6.23
6	48	Surgery	6.10
7	42	Cell Biology	5.34
8	36	Radiology Nuclear Medicine Medical Imaging	4.57
9	33	Hematology	4.19
10	30	Engineering Biomedical	3.81

The journal with the highest number of articles in this area of research was Frontiers in Cardiovascular Medicine (30), followed by Catheterization and Cardiovascular Interventions (28) and Atherosclerosis, Thrombosis, and Vascular Biology (23). The highest ACI values were found in the Journal of the American College of Cardiology (202.25), Circulation (93.23), and Atherosclerosis, Thrombosis, and Vascular Biology (69.87) (Table 5).

**TABLE 5:** Top 10 journals in the field of CAVS treatment research.

Rank	Journal	Quantity	ACI	STC
1	Frontiers in Cardiovascular Medicine	30	7.83	235
2	Catheterization and Cardiovascular Interventions	28	17.29	484
3	Arteriosclerosis, Thrombosis and Vascular Biology	23	69.87	1607
4	International Journal of Molecular Sciences	18	10.56	190
5	Journal of the American College of Cardiology	16	202.25	3236
6	Journal of Clinical Medicine	14	2.57	36
7	Circulation	13	93.23	1212
8	Journal of Thoracic and Cardiovascular Surgery	12	41.50	498
9	PLOS ONE	12	35.17	422
10	European Heart Journal	12	50.67	608

ACI, average citations per item.

### 3.5 Highly cited literature analysis

As shown in Table 6, the most cited article was “Two-Year Outcomes after Transcatheter or Surgical Aortic-Valve Replacement (Kodali et al., 2012).” Kodali et al. found, after at least 2 years of follow-up, that TAVR could be used as an alternative to surgery for high-risk patients.

**TABLE 6:** Top 10 cited articles in the field of valvular calcification stenosis treatment.

Rank	Article title	Journal	Type	Authors	Year	Citations	Country
1	Two-Year Outcomes after Transcatheter or Surgical Aortic-Valve Replacement	NEW ENGLAND JOURNAL OF MEDICINE	Original Article	Kodali et al.	2012	1833	United States
2	Intensive lipid lowering with simvastatin and ezetimibe in aortic stenosis	NEW ENGLAND JOURNAL OF MEDICINE	Original Article	Rossebo et al.	2008	1208	Norway
3	A randomized trial of intensive lipid-lowering therapy in calcific aortic stenosis	NEW ENGLAND JOURNAL OF MEDICINE	Original Article	Newby et al.	2005	808	United Kingdom
4	A Test in Context: Lipoprotein(a) Diagnosis, Prognosis, Controversies, and Emerging Therapies	JOURNAL OF THE AMERICAN COLLEGE OF CARDIOLOGY	Review	Tsimikas et al.	2017	664	United States
5	Calcific aortic stenosis	NATURE REVIEWS DISEASE PRIMERS	Original Article	Pibarot et al.	2016	583	Canada
6	Osteogenesis associates with inflammation in early-stage atherosclerosis evaluated by molecular imaging in vivo	CIRCULATION	Original Article	Aikawa et al.	2007	526	United States
7	Calcific Aortic Stenosis A Disease of the Valve and the Myocardium	JOURNAL OF THE AMERICAN COLLEGE OF CARDIOLOGY	Review	Dweck et al.	2012	467	United Kingdom
8	Aortic-Valve Stenosis - From Patients at Risk to Severe Valve Obstruction	NEW ENGLAND JOURNAL OF MEDICINE	Review	Otto et al.	2014	378	United States
9	Oxidized phospholipids are proinflammatory and proatherogenic in hypercholesterolaemic mice	NATURE	Original Article	Witztum et al.	2018	347	United States
10	Impact of Aortic Valve Calcification, as Measured by MDCT, on Survival in Patients With Aortic Stenosis	JOURNAL OF THE AMERICAN COLLEGE OF CARDIOLOGY	Original Article	Enriquez-Sarano et al.	2014	338	The Portuguese Republic

The second most cited article is “Intensive Lipid Lowering with Simvastatin and Ezetimibe in Aortic Stenosis”. In this article, Rossebo et al. confirmed after long-term follow-up and study that simvastatin and ezetimibe did not reduce the combined outcome of aortic valve events and ischemic events in patients with aortic stenosis (Rossebø et al., 2008).

The third most cited article, “A Randomized Trial of Intensive Lipid-Lowering Therapy in Calcific Aortic Stenosis,” authored by Cowell et al., presents findings from a cohort study indicating that intensive lipid-lowering therapy does not impede the progression or induce regression of calcific aortic stenosis (Cowell et al., 2005). Because calcified aortic stenosis shares many of the same characteristics with atherosclerosis (Pawade et al., 2019). Through research, we know that the process of atherosclerosis can be delayed by reducing the level of blood lipids in the body (Corti et al., 2001). Interestingly, this article presents a completely different conclusion and serves as a driving force for further long-term and larger-scale experiments on the potential use of statins in treating CAVS.

These papers furnish a valuable theoretical foundation and significant clinical insights for further research in this field. The ten most frequently cited documents included three reviews and seven original articles. The top three most cited articles are all original research papers, indicating that over the past 20 years, a large number of scholars have sought to conduct more in-depth research and analysis in this field, resulting in a significant volume of publications during this period.

### 3.6 Co-cited references analysis

**FIGURE 6:**
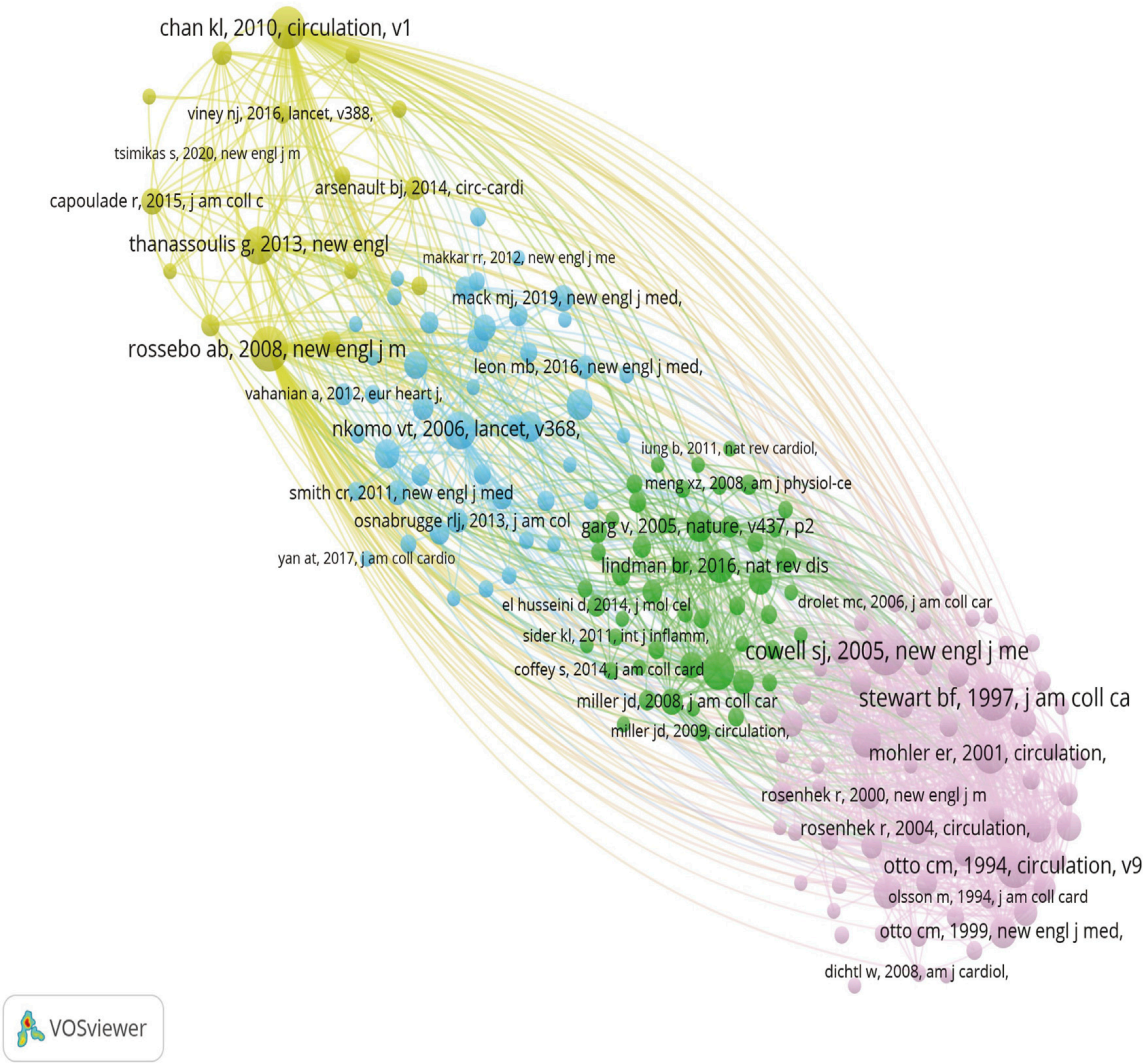
Different colors represent different clusters, larger circles represent more co-citation, and thicker lines between the two represent a stronger connection.

We analyzed 21,550 co-cited references, with the top ten listed in Table 7. The most frequently cited article, titled “A randomized trial of intensive lipid-lowering therapy in calcific aortic stenosis” from the New England Journal of Medicine, has been cited 181 times, underscoring its significant impact on the treatment of calcific aortic stenosis. Circulation, a leading journal in cardiovascular research, published five of the top ten articles, representing half of the most influential studies in this field. Among these ten articles, clinical drug trials, risk factors, and pathogenesis of valvular stenosis are prominent research hotspots. By clustering references with more than 20 co-citations, four distinct clusters were identified, as illustrated in Figure 6. These clusters represent key areas of focus within the literature, providing insights into the primary research directions and the evolving landscape of CAVS treatment.

**TABLE 7:** The top 10 co-cited articles in CAVS therapy exploration studies.

Rank	Title	Citation	Year	Author	Journal
1	A randomized trial of intensive lipid-lowering therapy in calcific aortic stenosis	181	2005	Newby et al.	NEW ENGLAND JOURNAL OF MEDICINE
2	Clinical factors associated with calcific aortic valve disease. Cardiovascular Health Study.	171	1997	Stewart et al.	Journal of the American College of Cardiology
3	Intensive lipid lowering with simvastatin and ezetimibe in aortic stenosis	154	2008	Rossebo et al.	NEW ENGLAND JOURNAL OF MEDICINE
4	Characterization of the early lesion of ‘degenerative’ valvular aortic stenosis. Histological and immunohistochemical studies.	152	1994	Otto et al.	Circulation
5	Effect of Lipid Lowering With Rosuvastatin on Progression of Aortic Stenosis Results of the Aortic Stenosis Progression Observation: Measuring Effects of Rosuvastatin (ASTRONOMER) Trial	132	2010	Chan et al.	Circulation
6	Bone formation and inflammation in cardiac valves	114	2001	Mohler et al.	Circulation
7	Burden of valvular heart diseases: a population-based study	110	2006	Nkomo et al.	LANCET
8	Calcific Aortic Valve Disease: Not Simply a Degenerative Process A Review and Agenda for Research From the National Heart and Lung and Blood Institute Aortic Stenosis Working Group	109	2011	Rajamannan et al.	Circulation
9	Genetic Associations with Valvular Calcification and Aortic Stenosis	105	2013	Thanassoulis et al.	NEW ENGLAND JOURNAL OF MEDICINE
10	Human aortic valve calcification is associated with an osteoblast phenotype	94	2003	Rajamannan et al.	Circulation

### 3.7 Hotspots and future directions

#### 3.7.1 Analysis of research hotspots

Keywords serve as the condensed knowledge network encapsulating the essence of a research paper, thereby facilitating the identification of frontier changes in related research. As shown in Table 8, except for “stenosis” and “calibration”, the keywords with the highest frequency are “progression” (160), “disease” (158), “aortic stenosis” (153), “inflammation” (117), “aortic valve stenosis” (115) and so on. These represent some hot spots in CAVS research and potential targets in future treatment.

**TABLE 8:** Top 10 keywords in CAVS treatment research.

Rank	Keyword	Occurrence	Total link strength
1	Calcification	295	2156
2	Stenosis	294	2087
3	Progression	160	1345
4	Disease	158	1151
5	Aortic stenosis	153	1117
6	Inflammation	117	962
7	Aortic valve stenosis	115	893
8	Association	101	844
9	Replacement	88	598
10	Expression	86	646

**FIGURE 7:**
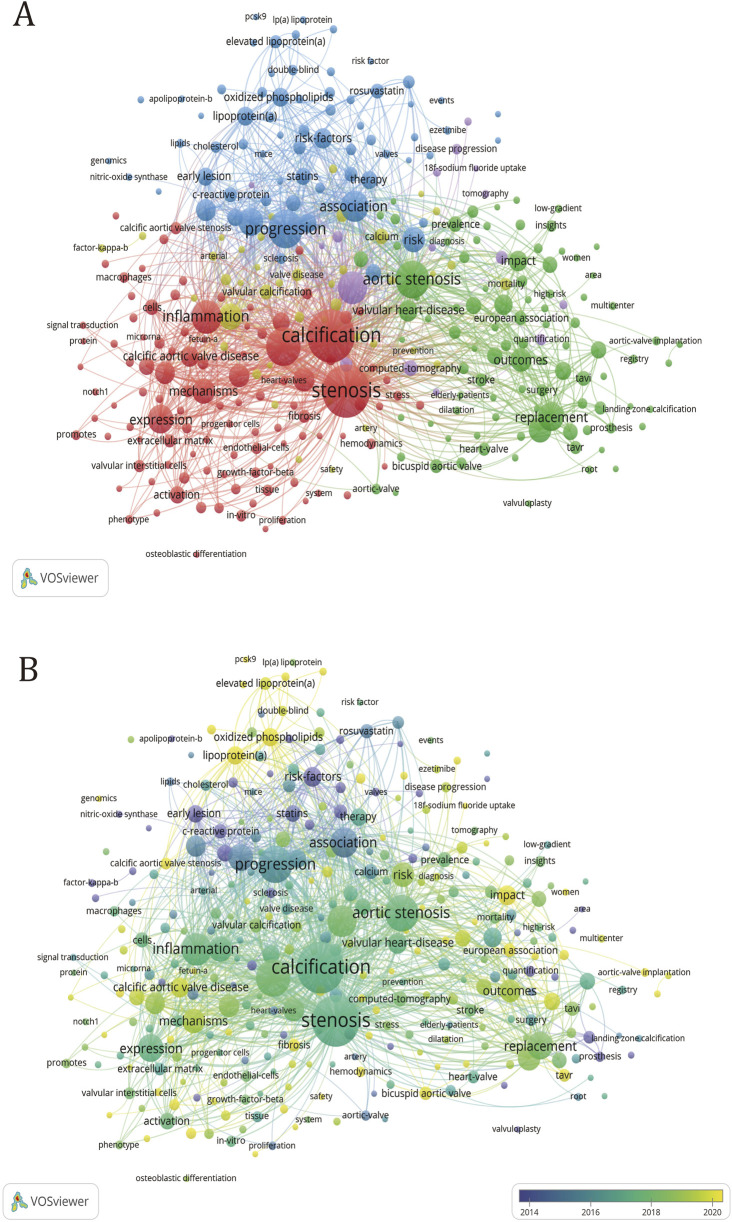
Keyword clustering map in CAVS treatment research. **(A)** The size of the circle is proportional to the number of times the keyword appears. The degree of proximity of the circles indicates the frequency of co-occurrence between the two corresponding keys, and the closer they are, the higher the degree of co-occurrence between them. **(B)** Added timeline to **(A)**.

The co-occurrence network of keywords depicted in Figure 7A delineates the interconnections between various terms within the research domain. The thickness of lines connecting nodes corresponds to the frequency of occurrence of paired keywords. This visualization reveals five distinct clusters of keywords, each representing a unique direction within the research field. To this, we have added a timeline (Figure 7B), where the yellow color represents the more frequent keywords in relatively recent years, while the purple color represents the keywords that appeared earlier. The addition of the timeline helps to analyze which research directions have been hot over the years.

#### 3.7.2 Green cluster

Within the green cluster, the keyword “aortic stenosis” emerges with the highest frequency of occurrence and exhibits the most frequent connections with other keywords. From a clinical perspective, most patients diagnosed with valvular calcification exhibit varying degrees of aortic stenosis. Articles with the keyword “aortic stenosis” examined the number of patients, epidemiological characteristics, diagnosis, and treatment methods to characterize the diagnosis and treatment of aortic calcification (Pawade et al., 2019). The papers under the keyword “management” examined the epidemiological characteristics and looked at prevention, medical therapy, transcatheter valve replacement, and comparing CT and echocardiography to diagnose the disease (Bonow et al., 2016). The paper on “Sex differences” examines the pathophysiological processes, molecular differences and imaging differences in valvular calcification between men and women to see if there is a potential for individualized treatment options based on sex (Desjardin et al., 2022). The paper with the keyword “bicuspid aortic valve” discusses the comparison of outcomes after TAVR surgery for calcific aortic stenosis and variant valve interventions (Lindman et al., 2016; Moncla et al., 2023). The “multicentre” article presents the results of many multicentre clinical trials, which provide a good reference for subsequent diagnosis, treatment and management. After adding the timeline, we can see that “sex difference”, “management” and “impact” are all high-frequency keywords. The keywords also give us new perspectives on prevention, treatment and management.

#### 3.7.3 Red cluster

In the red cluster, the keyword with the highest frequency of occurrence is “stenosis”, but the keyword with the most links to other keywords is “calcification”. The composition of the keywords shows that most of the keywords in this group describe calcification and stenosis from the perspective of molecular mechanisms. Papers with the keyword “cell” discussed aortic valve interstitial cells and aortic valve endothelial cells (Butcher and Nerem, 2007). The former is stimulated by basement membrane destruction, lipoprotein accumulation, oxidative stress, and inflammatory cell infiltration to differentiate into osteoblasts (Yetkin and Waltenberger, 2009; Mohty et al., 2008; Natorska et al., 2021), and the latter switch to interstitial cells after being stimulated by shear forces (Zhong et al., 2023). Besides, it can also promote the osteogenic differentiation of interstitial cells. Papers with the keyword “interstitial cell” studied the dysfunction of the Notch (Nigam and Srivastava, 2009; Yutzey et al., 2014), and Wnt (Zhou et al., 2019) pathways, and the dysregulation of microRNA (Zeng et al., 2021; Jiao et al., 2019) resulting in increased bone signaling in valve interstitial cell (VIC), leading to osteogenic differentiation. Papers with the keyword “inflammation” studied that the inflammatory infiltration in the calcified aortic valve was composed of macrophages (Otto et al., 1994; Li et al., 2017), mast cells (Otto et al., 1994), CD4^+^ T cells, and CD8^+^ T cells, which proved that the remodeling of aortic valve cells was related to inflammation (Passos et al., 2020). Papers with the keyword “autophagy” demonstrated that short-term enhancement of autophagy and mitochondrial autophagy reversed the CAVS phenotype, suggesting that impaired mitochondrial quality and dysregulated autophagy exacerbate the progression of CAVS (Morciano et al., 2022). “VICs,” “valve endothelial cells (VECs)” and “mechanisms” have all been hot topics of research in the last few years, which proves that the current research on the molecular mechanisms of CAVS is still hot, research is still hot.

#### 3.7.4 Yellow cluster

In the yellow cluster, the keyword that appears most frequently is “aortic valve stenosis” and has the most links with other keywords. Papers with the keyword “Risk factor” investigate the role of high-density lipoprotein, oxidized phospholipids, and Lp(a) in elucidating the deposition of lipogenic substances as one of the key risk factors contributing to CAVS (Myasoedova et al., 2018; Graham et al., 2016), it also clarified that oxidative stress, inflammation, and coronary artery disease are all risk factors for aortic valve calcification (Cho et al., 2018). Papers with the keyword “matrix-gal protein (MGP),” suggest that MGP inhibits tissue calcification and that there may be various potential pathways by which MGP gene expression could regulate tissue calcification, and even serve as a genomic biomarker for predicting the progression of calcification (Bjørklund et al., 2020). The paper with the keyword “chronic kidney disease” reveals that the presence of chronic kidney disease is an important risk factor for valvular stenosis, leading to accelerated valvular calcification (Pan et al., 2023; Hutcheson and Goettsch, 2023). From the composition of keywords in this group, most of them are risk factors that can accelerate valve calcification, among which “chronic kidney disease” and “metabolism” are popular research directions that have emerged in the past 5 years.

#### 3.7.5 Blued cluster

In the blue cluster, the keyword with the most frequent occurrence is “progress”, and it also has the most links with other keywords. Papers with the keyword “atherosclerosis” have studied that there are many similarities between atherosclerosis and calcific aortic stenosis, mainly involving lipid accumulation and calcium deposition (Stewart et al., 1997; Sá et al., 2020; Mazzone et al., 2007). The paper with the keywords “oxidized phospholipid” examined whether lowering circulating levels of lipoproteins and oxidized phospholipids with statins would slow the progression of calcific aortic stenosis (Que et al., 2018). Papers with the keyword “low-density lipoprotein” examined the close association of lipoprotein deposition with both the onset and progression of atherosclerosis, aortic valve calcification, and arterial stenosis (Seo et al., 2023). Papers with the keyword “Lp(a)” investigated that high circulating levels of Lp (a) promote the accumulation of Ox-PL in the aortic valve (Kronenberg and Utermann, 2013; Wiesner et al., 2013), which in turn leads to the production of calcification. Papers with the keyword “familial hypercholesterolemia” have studied the key role of LDL-C metabolism in Cavs etiology (Rajamannan, 2015; Ten et al., 2015). Papers with the keyword “metabolism” discuss obesity-related metabolic syndrome as a powerful predictor of aortic stenosis (Capoulade et al., 2012), in addition, the high level of blood lipids in the body caused by metabolic syndrome promotes osteogenic differentiation of VICs (Perrot et al., 2020). Meanwhile, the papers with the keyword “hypoxia” investigated that hypoxia promotes the development of CAVS by causing chronic inflammation and abnormalities in lipid metabolism (Bouhamida et al., 2023). Most of the keywords in this collection are related to lipid metabolism, while “Lp(a),” “PCSK9,” and “oxidized phospholipid” are all hot research topics today.

#### 3.7.6 Purple cluster

In the purple collection, the keyword with the most frequent occurrence is “computed tomography” and has the most connections with other keywords. Papers with the keyword “computed tomography” examine computed tomography (CT) as an effective method of measuring aortic valve calcification as a function of stenosis severity and disease progression (Dweck et al., 2023; Otto et al., 2021; Vahanian et al., 2021). The article entitled “18F-Sodium Fluoride (18F-NaF)” sought to determine whether aortic stenosis and atherosclerosis exhibit analogous markers that can be employed to predict disease severity (Tzolos and Dweck, 2020). The article entitled “Coronary Artery Calcium” examines the similarities between calcific aortic stenosis and atherosclerosis in early-stage disease, as well as the potential applications of novel imaging techniques for quantifying calcium levels (Pan et al., 2023). After adding the timeline, it is evident that the use of 18F-NaF PET and computed tomography for diagnosis and treatment has been a hot research topic in recent years. At the same time, it can also be found that most of the keywords in the purple cluster appear relatively late and are currently a hot research area.

### 3.8 Recognition of cutting-edge research in the literature

All keywords that appeared between January 2003 and September 2024 were subjected to citation bursting through Citespace to determine the 20 keywords with the highest citation bursting rate, which are presented in Table 9. The year column denotes the year in which the keyword first appeared. The Begin and End columns denote the year in which the keyword was first identified as a frontier and the year in which it ceased to be identified as a frontier, respectively. The Strength column denotes the strength of the bursting. As illustrated in Table 9, citation burst terms such as “risk factor,” “hypercholesterolemia,” “association,” and “mechanisms” emerged as early research frontiers in 2003, reflecting initial priorities in the study of CAVS. Concurrent bursts of interest in terms like “C-reactive protein,” “statins,” “heart valve,” and “valvular calcification” further highlight the field’s focus on inflammation, lipid metabolism, and structural changes in the aortic valve. In recent years, burst terms such as “impact,” “valvular interstitial cells,” and “mechanisms” have remained prominent, reflecting sustained research interest in understanding the pathogenesis of CAVS and identifying innovative therapeutic targets.

**TABLE 9:** Top 20 keywords with the strongest citation bursts.

Keywords	Year	Strength	Begin	End	2003–2024
Early lesion	2005	10.08	2005	2014	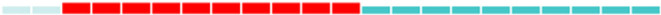
Risk factors	2003	5.39	2003	2014	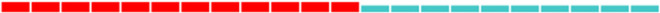
Association	2003	5.39	2003	2014	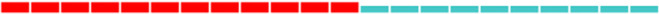
Hypercholesterolemia	2003	4.36	2003	2011	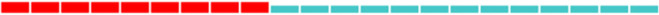
Angiotensin-converting enzyme	2006	8.68	2006	2014	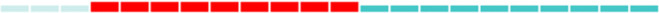
C reactive protein	2006	7.9	2006	2011	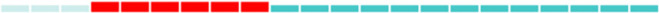
Statins	2006	4.73	2006	2011	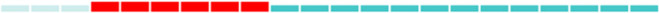
Randomized trial	2006	4.42	2006	2014	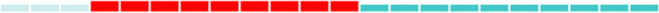
Inhibitors	2006	4.4	2006	2011	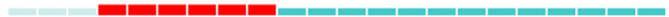
Rosuvastatin	2010	6.98	2010	2014	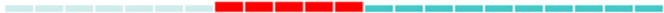
High risk patients	2009	5.24	2009	2017	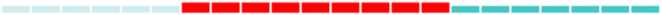
Prosthesis	2009	4.59	2009	2014	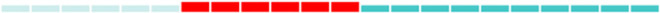
Transcatheter aortic valve implantation	2009	4.21	2009	2017	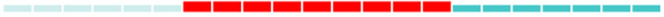
Heart valve	2009	4.68	2015	2020	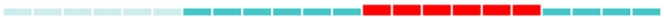
Valvular calcification	2015	4.2	2015	2020	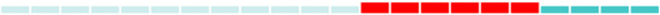
European association	2018	4.56	2018	2024	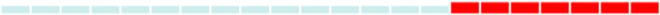
Cardiovascular disease	2016	4.31	2018	2023	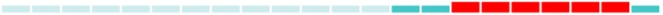
Impact	2015	4.04	2018	2024	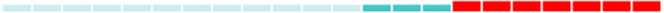
Valvular interstitial cells	2009	5.07	2021	2024	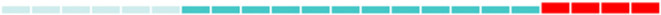
Mechanisms	2003	4.4	2021	2024	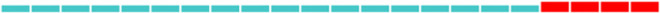

This analysis not only highlights evolving trends in the literature but also underscores the dynamic nature of CAVS research, shifting from risk factor identification to deeper investigations into molecular mechanisms and innovative treatment approaches.

## 4 Discussion

The present study conducted a bibliometric analysis of literature spanning from 2003 to 2024, focusing on the treatment of aortic stenosis using CiteSpace and VOSviewer software. By examining spatial and temporal distribution, regional cooperation, author contributions, core literature, hot topics, and research frontier analysis through keyword co-occurrence analysis, we identified emerging research themes across different periods and elucidated the evolution path of this research field. Additionally, we pinpointed the forefront of current research on the treatment of CAVS. The findings of this bibliometric analysis provide valuable insights into the development of treatment strategies for aortic stenosis, offering a comprehensive overview of emerging trends and key research areas that might guide future investigations and clinical advancements in the field.

### 4.1 The research on the treatment of CAVS shows an uneven upward trend

Research on the treatment of CAVS has shown a fluctuating upward trend over time. The number of publications on CAVS treatment has steadily increased, culminating in 2022, indicating that this field remains a prominent research hotspot. Additionally, citations related to CAVS treatment have risen consistently, showing a near-linear increase from 2019 to 2022. These trends reflect the continued focus and acknowledgment of this field within the academic community.

CAVS research originated in developed countries, with highly cited articles predominantly produced by institutions in these regions. Leading institutions such as Harvard University, Laval University, and the University of California rank among the top three in publication volume. Harvard University, in particular, holds the highest position and has delved into more intricate research directions. Overall, Western developed countries excel in the quality and quantity of CAVS research.

The reasons behind these disparities between developed and less-developed countries can be attributed to several key factors. First, treatment modalities and resource constraints: CAVS treatment primarily involves surgery and interventional therapies, which are challenging to implement widely in low-income countries due to technological and financial limitations. Second, population structure and prevalence: longer life expectancies and lifestyle differences in developed countries contribute to a higher prevalence of CAVS, creating greater demand for research. Third, policy support: developed countries recognize CAVS as a critical area of chronic disease research and benefit from favorable policies, whereas resource-limited countries prioritize infectious diseases and basic healthcare, providing less support for CAVS-related studies.

From a regional perspective, Western countries have more advanced and comprehensive approaches to CAVS treatment than those in Asia and other developing regions. Notably, China, a developing country and a leading Asian contributor, ranks third globally in publication volume in this field, reflecting the growing emphasis on CAVS treatment in developing and Asian countries. However, among the top ten institutions by publication volume, six are based in the U.S., and two in Canada, with no Asian institutions represented. Furthermore, while European countries collaborate closely within the region, other parts of the world exhibit relatively less international cooperation. To advance the field of CAVS treatment, fostering global collaboration, leveraging regional strengths, and promoting mutual benefits are essential.

Interdisciplinary collaboration is essential for advancing CAVS research. Related disciplines, such as cardiology, cell biology, internal medicine, pharmacology, and surgery, provide complementary expertise to foster innovative treatment strategies. For instance, the integration of pharmacology and cell biology enables the exploration of novel therapeutic targets, while the collaboration between materials science, physiology, and surgery facilitates the development of improved bioprosthetic valves. Such efforts ensure diverse perspectives and drive breakthroughs in CAVS treatment.

Journal classifications and impact factors remain essential indicators of academic quality. Papers published in Q1 or high-impact factor journals typically garner greater credibility and influence. In the CAVS field, highly cited and co-cited papers are predominantly published in Q1 journals, highlighting the substantial academic value of the field and the robustness of its findings. The most frequently cited documents focus primarily on clinical trials and mechanistic studies, including comparisons of surgical and interventional treatments, lipid-lowering therapies, risk factors, inflammation, and oxidized phospholipids.

In conclusion, research on CAVS treatment continues to prioritize technological innovations, clinical drug trials, and the exploration of novel targets and mechanisms. While highly cited literature serves as an essential reference in the field, citation counts alone are insufficient to fully reflect the quality of a paper. Early-published papers often accumulate more citations over time. Therefore, evaluating article quality should incorporate innovation and scientific contributions rather than relying solely on citation numbers.

### 4.2 The hotspots and frontiers

Keyword analysis is a valuable tool for gaining insights into the CAVS research field, revealing the distribution of research priorities and emerging trends. Therapeutic strategies need to be tailored to the patient’s pathological state and individual circumstances. For patients with early-stage CAVS, pharmacological treatments may help delay disease progression, whereas patients with advanced disease typically require TAVR or SAVR to alleviate symptoms and improve survival. Studies have demonstrated that TAVR significantly reduces mortality and enhances quality of life in both high- and intermediate-risk patients. However, some advanced-stage patients highlight the need for further optimization of treatment strategies, as they may be ineligible for invasive procedures or face risks such as postoperative restenosis and valve failure. Cluster analysis of keywords and timeline studies has revealed that the core directions of CAVS treatment over the past 20 years include the evolution of therapeutic concepts, advancements in diagnostic methods, exploration of etiological mechanisms, enhancement of prognostic management, and the development of novel drug targets. These changes reflect a shift from monotherapy to integrated management and from traditional approaches to innovative strategies.

#### 4.2.1 Shift in therapeutic philosophy

With advancing research into valvular stenosis, treatment concepts for patients with this condition have significantly evolved. While traditional strategies primarily targeted symptomatic patients, current approaches advocate for broader treatment scopes, including early intervention. Some studies recommend intervention at the earliest signs of calcification to slow disease progression and improve long-term outcomes. Mendelian randomization and observational studies have identified obesity as a major risk factor for CAVD (Moncla et al., 2023). Meanwhile, the American Heart Association (AHA) has identified seven key factors influencing cardiovascular health—BMI, healthy diet, physical activity, smoking, blood pressure, diabetes, and total cholesterol (Lloyd-Jones et al., 2010). Therefore, it is crucial to promote healthy lifestyles and risk-reducing medical awareness among the general public and early-stage patients, as this can effectively prevent the onset and progression of CAVS. By addressing these risk factors early, the need for invasive treatments in advanced stages of the disease can be significantly reduced, ultimately improving patients’ long-term prognosis and quality of life.

#### 4.2.2 Innovations in diagnostic methods

Currently, patients’ valvular stenosis diagnosis is primarily based on symptoms and echocardiography. However, echocardiography cannot directly detect the presence of valve calcification (Tastet et al., 2024). With the advancement of emerging technologies, CT scans—especially low-dose and dual-source CT—have greatly improved diagnostic accuracy and efficiency. Low-dose CT reduces radiation exposure while maintaining high-resolution imaging, which is crucial for ongoing monitoring in high-risk patients (Hausleiter et al., 2010). Dual-source CT, which uses two X-ray sources, enables faster and more precise image acquisition, which is particularly beneficial for patients with higher heart rates (Flohr et al., 2006). It also allows for accurate measurements of the annular area and enhanced visualization of valve calcification. These improvements help better assess patient conditions and plan treatments with greater precision, especially for procedures like TAVR. Additionally, multidetector CT (MDCT) offers reliable calcification scoring, utilizing systems such as the Agatston and volume scores, with gender-specific thresholds to provide tailored assessments (Tzolos et al., 2022; Budoff et al., 2006). Guidelines currently recommend this method for evaluating patients with inconclusive echocardiographic results. Functional imaging methods, including 18F-NaF PET, assess calcification activity, facilitating disease monitoring and risk prediction (Tzolos and Dweck, 2020; Tzolos et al., 2020). These technologies are highly valuable for early detection of disease progression, particularly in high-risk patients, and can also guide treatment decisions. Moreover, biomarkers such as hs-TnI and hs-CRP (Iglesias-álvarez et al., 2018), linked to inflammation and cardiovascular risk, and IL-6 (Junco-Vicente et al., 2022), associated with calcification progression, offer the potential for assessing disease severity, evaluating risks, and predicting the prognosis. By integrating advanced imaging technologies with biomarkers, a more comprehensive approach to diagnosing and managing valvular stenosis can be adopted, leading to personalized treatment strategies and improved patient outcomes.

#### 4.2.3 Exploration of new mechanisms

Mechanistic studies of CAVS have intensified in recent years, expanding beyond traditional mechanisms such as chronic inflammation, osteogenic differentiation, lipid deposition, and mechanical stress. Emerging research has uncovered novel pathways, including mitochondrial dysfunction and dysregulated autophagy. A recent study by Morciano et al. demonstrated that mitochondrial turnover is dysregulated in CAVS patients, leading to impaired mitochondrial function and autophagy (Morciano et al., 2022). Notably, rapamycin was shown to reverse the calcification phenotype, suggesting that enhancing autophagy and mitophagy could be an effective therapeutic strategy. This finding provides a theoretical foundation for developing drugs targeting mitochondrial quality and autophagy. Additionally, cellular senescence has been identified as a key feature of CAVS, with senescent VIC exhibiting pro-inflammatory and pro-calcific phenotypes driven by the secretion of senescence-associated secretory phenotype (SASP) factors (Phadwal et al., 2021). Targeting cellular senescence may therefore help delay or reverse valve calcification, particularly in aging populations. Earlier studies have also highlighted the role of hypoxia in CAVS progression, showing that thickened valves in early-stage CAVS impair oxygen delivery, while persistent hypoxic stimuli exacerbate inflammation and mitochondrial dysfunction, further promoting calcification. These findings confirm that hypoxia signaling pathways play a critical role in both the early and advanced stages of CAVS (Bouhamida et al., 2023; Molnár et al., 2022). Improving oxygenation or targeting hypoxic signaling pathways may thus offer therapeutic benefits. Collectively, these insights provide new directions for the individualized treatment of CAVS, enabling clinicians to develop precise therapeutic strategies by considering patient-specific factors and leveraging advances in drug development and research.

#### 4.2.4 Emerging therapeutics for lowering Lipoprotein(a) and mitigating CAVS progression

Pharmacological treatment remains a central focus in the management of CAVS patients, with higher patient acceptance compared to invasive procedures like TAVR or SAVR. Over the past two decades, researchers have explored various drugs to slow the progression of CAVS, with clinical trials involving statins, vitamin K2, and warfarin showing limited success. Lipoprotein(a) [Lp(a)] is a low-density lipoprotein particle covalently bound to an apolipoprotein(a) [Apo(a)] moiety, which is attached to its apolipoprotein B (ApoB) component (O'Donoghue et al., 2022). Accumulating evidence indicates that Lp(a) is a strong, independent, and potentially causal risk factor for CAVS (Duarte and Giugliano, 2022; Kamstrup et al., 2014). To date, Lp(a) remains the only monogenetic risk factor identified for CAVS (Thanassoulis, 2016). Elevated Lp(a) levels have been associated with an increased incidence of aortic valve calcification, accelerated hemodynamic progression of aortic stenosis, and a greater need for aortic valve replacement, particularly in younger patients (Guddeti et al., 2020). In recent years, significant advancements have been made in the development of pharmacological strategies aimed at reducing Lp(a) levels and potentially attenuating CAVS progression (Tsamoulis et al., 2023). Among these, RNA-targeting therapies have emerged as a novel and promising approach, utilizing intracellular pathways to suppress Lp(a) synthesis through gene silencing mechanisms (Kosmas et al., 2023). Two key categories of oligonucleotide-based gene silencing agents are currently under investigation: antisense oligonucleotides (ASOs) and small interfering RNAs (siRNAs) (Khan et al., 2024; Duarte and Giugliano, 2022).

##### 4.2.4.1 Antisense oligonucleotide therapy: pelacarsen

Pelacarsen (IONIS-APO(a)-LRx) is an advanced ASO-based therapeutic agent derived from IONIS-APO(a)Rx, featuring a triantennary N-acetyl-galactosamine (GalNAc) conjugation to facilitate targeted hepatic uptake via the asialoglycoprotein receptor (Tsimikas et al., 2015). In a phase II multicenter trial involving 286 patients with established cardiovascular disease (CVD) and baseline Lp(a) levels ≥150 nmol/L, pelacarsen demonstrated a dose-dependent reduction in Lp(a) levels, with reductions of up to 80% compared to placebo at 6 months (Tsimikas et al., 2020; Tsimikas et al., 2021). The Lp(a)HORIZON trial, an ongoing phase III, randomized, placebo-controlled study, is evaluating the clinical efficacy and safety of monthly subcutaneous pelacarsen (80 mg) in patients with established CVD and Lp(a) ≥70 mg/dL. This trial has enrolled 8,221 patients and is anticipated to be completed in 2025 (ClinicalTrials.gov Identifier: NCT04023552).

##### 4.2.4.2 Small interfering RNA (siRNA) therapies

Following the development of ASOs, siRNA-based therapies have emerged as another promising strategy to directly suppress Lp(a) production by inhibiting apo(a) mRNA translation. Several siRNA therapeutics are currently undergoing clinical evaluation:

###### 4.2.4.2.1 SLN360

SLN360 is a GalNAc-conjugated siRNA designed to silence LPA mRNA, thereby reducing hepatic Lp(a) synthesis. The phase I APOLLO trial enrolled 32 participants without known CVD but with baseline Lp(a) levels ≥150 nmol/L, randomizing them to receive a single dose of SLN360 or placebo. The results demonstrated a dose-dependent, sustained reduction in Lp(a), with the highest dose (600 mg) achieving up to a 98% reduction (Nissen et al., 2022). A multicenter, randomized, double-blind, placebo-controlled phase II trial has completed patient enrollment as of July 2024, and further clinical outcome data are awaited (ClinicalTrials.gov Identifier: NCT05537571).

###### 4.2.4.2.2 Olpasiran

Olpasiran (AMG 890) is an siRNA therapeutic specifically designed to target LPA mRNA and suppress Lp(a) production (O'Donoghue et al., 2022). The phase II OCEAN(a)-DOSE trial demonstrated a robust, dose-dependent reduction in Lp(a) levels, with sustained effects observed nearly 1 year after a single subcutaneous administration (O'Donoghue et al., 2024). The phase III OCEAN(a) Outcomes Trial is currently enrolling 6,000 patients to assess the impact of Olpasiran on cardiovascular outcomes and Lp(a) reduction, with completion anticipated by the end of 2026 (ClinicalTrials.gov Identifier: NCT05581303).

###### 4.2.4.2.3 LY3819469

LY3819469 is a next-generation GalNAc-conjugated siRNA therapy targeting LPA mRNA for Lp(a) reduction. A phase I clinical trial enrolled 66 participants with elevated Lp(a) levels and increased CVD risk (Sheridan, 2022). Preliminary data suggest that LY3819469 exhibits potent Lp(a)-lowering effects, supporting its potential as a promising therapeutic candidate for further clinical investigation.

Simultaneously, histone deacetylase (HDAC) inhibitors have shown potential in preclinical studies by epigenetically regulating osteogenic differentiation in valvular interstitial cells (Gu et al., 2019), but concerns about side effects, specificity, and the lack of large-scale clinical trial data limit their development. To enhance drug targeting, Wang et al. combined magnetic targeting with nanocarriers to deliver the anticoagulant XCT790 directly to calcified aortic valves, significantly reducing calcification and stenosis in mouse models (Chen et al., 2024), offering a novel therapeutic direction despite challenges in clinical translation due to technical complexity and cost. Additionally, inositol hexaphosphate hexasodium salt (SNF472), a vascular calcification inhibitor that blocks calcium-phosphate crystal formation (Zabirnyk et al., 2020; Zabirnyk et al., 2019), has shown promise in early clinical trials by slowing arterial calcification progression, though its application in CAVS requires further exploration (Raggi et al., 2020). Collectively, these innovative therapies mark a transformative shift in CAVS treatment, but their successful translation hinges on overcoming critical challenges in drug delivery, safety, and clinical validation.”

#### 4.2.5 Enhanced postoperative management

Post-surgery follow-up leverages three-dimensional ultrasound and CT scans to evaluate valve function and calcification progression, facilitating timely detection of recalcification and stenosis (D'Humières et al., 2018). Meanwhile, with the widespread adoption of digital health, devices can be worn to monitor patients’ blood pressure, heart rate, ECG, etc., in real-time, enabling better assistance for physicians in adjusting treatment plans and reducing rehospitalization rates. In the future, leveraging insights from CAVS pathological mechanisms—such as hypoxia signaling pathways, cellular senescence, and mitochondrial dysfunction—researchers can further develop biomarker-based monitoring tools. These tools, which may include blood tests or imaging techniques to detect SASP factors or assess mitochondrial function, could enable early warning and intervention. Such a multidimensional and individualized prognostic management strategy would not only enhance patients’ quality of life but also provide robust support for the precision treatment of CAVS.

Recent progress in CAVS research has introduced advanced diagnostic tools, such as low-dose CT, dual-source CT, and biomarkers like IL-6, alongside innovative therapies, including RNA-based drugs and HDAC inhibitors. These advancements have enhanced diagnostic accuracy and broadened treatment possibilities, enabling personalized approaches that target mechanisms like mitochondrial dysfunction and cellular senescence. Additionally, digital health tools have improved postoperative monitoring. Although early findings indicate potential benefits in slowing disease progression and reducing the need for invasive procedures, the direct effects of these technologies on patient outcomes-such as survival symptom alleviation, and long-term quality remain insufficiently studied. Future research should prioritize longitudinal clinical validation to evaluate their real-world effectiveness and refine comprehensive CAVS management strategies.

### 4.3 Cooperation among states should be sustained

Regional differences play a significant role in CAVS research and treatment development, with North America and Europe leading in TAVR technology and multicenter clinical trials (Pan et al., 2023), while Asia has made substantial contributions to understanding genetic and molecular mechanisms. In contrast, resource-limited regions often struggle to access advanced diagnostic tools and treatments, highlighting the need for global collaboration to address these disparities. Establishing international multi-omics and AI databases to facilitate data sharing and technology transfer could provide critical support to these regions (Rehm et al., 2021). Advances in AI and diagnostic technologies will enable earlier identification of CAVS patients, promote more individualized treatment strategies, reduce societal burdens, and improve patients’ quality of life and long-term prognosis, particularly in resource-limited settings (Topol, 2019). Additionally, the application of AI in image analysis, predictive modeling, and drug screening can significantly enhance diagnostic accuracy and efficiency while accelerating the development of novel therapies. However, despite AI’s powerful capabilities in integrating information with remarkable efficiency and precision, it is essential to develop standardized data guidelines and strengthen ethical regulations (Price and Cohen, 2019). These measures will ensure that AI applications align with international norms, prevent unauthorized data use, and safeguard transparency and patient privacy.

### 4.4 Future direction

Bibliometrics comprehensively reveals the core issues and future directions in CAVS research by systematically analyzing research trends, hot areas, collaborative networks, and high-impact literature. For example, high-frequency keywords such as Lp(a) and calcification mechanisms reflect current research priorities, which are closely aligned with clinical needs. However, bibliometric analyses also highlight emerging research hotspots. (1) Significant gender differences in CAVS pathophysiology influence disease progression, clinical presentation, and treatment responses. Studies suggest that female patients typically exhibit valvular fibrosis and left ventricular diastolic dysfunction, while male patients tend to have more severe calcification and faster disease progression (Shames and Gillam, 2013), likely due to sex hormones’ differential effects on inflammation and osteogenic differentiation. This insight suggests that male patients may benefit from drugs targeting osteogenic differentiation and inflammation, whereas female patients may respond better to combinations of antifibrotic agents and cardiac function-improving drugs. Additionally, optimizing gender-specific imaging assessment criteria could enhance personalized therapeutic strategies. (2) Mitochondrial dysfunction and dysregulated autophagy have emerged as novel mechanisms driving valve calcification, with studies showing that rapamycin-induced autophagy activation can reverse many CAVS phenotypes (Morciano et al., 2022). However, further exploration is needed to bridge the gap between animal experiments and clinical efficacy. Improvements in preclinical models and multi-center randomized controlled trials (RCTs) are essential to evaluate the safety and efficacy of such therapies. (3) Prolonging the durability of prosthetic valves remains a critical research focus. Combining AI and advanced imaging technologies with 3D printing could enable the creation of patient-specific valve scaffolds (Ooms et al., 2021), while developing anti-inflammatory biological valves may reduce post-implantation inflammatory responses. Achieving these goals will require sustained global collaboration to advance the diagnosis, treatment, and prevention of CAVS.

### 4.5 Study limitations

Although the present study offers some insights into the evolution of CAVS treatment over the past two decades, several limitations should be acknowledged. First, to ensure data quality and analytical consistency, we selected the WoS database. It is worth noting that relying solely on the WoS database may limit the scope of the literature, as other relevant studies may be indexed in different databases, such as Scopus, potentially introducing selection bias. Additionally, using other databases might yield slightly different results. Literature searching together with other high-quality databases such as PubMed, and Scopus might minimize selection bias. However, bibliometrics cannot screen and sort the data of the given databases at the same time, searching multiple databases simultaneously might lead to a large number of duplicates, resulting in problems in subsequent analysis, so only WoS database is selected for current analysis. Since WoS database is a globally recognized and one of the most comprehensive resources linked with high quality with sufficient authoritative data for scholarly information, which helps minimize the impact of this limitation on our findings. Second, our analysis was limited to English-language publications, potentially omitting regionally specific treatments described in other languages. Third, technical challenges during data analysis, such as handling special characters in author or institution names, occasionally led to inaccuracies or inconsistencies in the dataset. Despite implementing corrective measures, such as standardizing character encoding and verifying the accuracy of author and institution information, some residual errors may have slightly affected the analysis, particularly in large datasets with multiple entries.

## 5 Conclusion

This study provides the first bibliometric analysis of global literature on treating CAVS spanning nearly 20 years. Utilizing visual analysis technology, we evaluated treatment progress, identified research hotspots, and proposed future directions. The present analysis indicates that the United States and Canada have been at the forefront of research on CAVS, with institutions and scholars from these developed countries playing a pivotal role in advancing the field. Over the past two decades, developing countries such as China have gradually intensified their research efforts on CAVS. Looking ahead, we anticipate that future research will further elucidate the evolution of treatment strategies, explore innovations in diagnostic and therapeutic technologies, deepen our understanding of the underlying mechanisms of CAVS, and identify novel pharmacological targets to halt or prevent CAVS progression.

## Data Availability

The original contributions presented in the study are included in the article/supplementary material, further inquiries can be directed to the corresponding author.
